# Lung Ultrasonography in the Evaluation of Late Sequelae of COVID-19 Pneumonia—A Comparison with Chest Computed Tomography: A Prospective Study

**DOI:** 10.3390/v16060905

**Published:** 2024-06-03

**Authors:** Katarzyna Zimna, Małgorzata Sobiecka, Jacek Wakuliński, Dorota Wyrostkiewicz, Ewa Jankowska, Monika Szturmowicz, Witold Z. Tomkowski

**Affiliations:** 1 I Department of Lung Diseases, National Tuberculosis and Lung Diseases Research Institute, 01-138 Warsaw, Poland; 2 Department of Radiology, National Tuberculosis and Lung Diseases Research Institute, 01-138 Warsaw, Poland

**Keywords:** COVID-19, chest computed tomography, lung ultrasonography

## Abstract

The onset of the COVID-19 pandemic allowed physicians to gain experience in lung ultrasound (LUS) during the acute phase of the disease. However, limited data are available on LUS findings during the recovery phase. The aim of this study was to evaluate the utility of LUS to assess lung involvement in patients with post-COVID-19 syndrome. This study prospectively enrolled 72 patients who underwent paired LUS and chest CT scans (112 pairs including follow-up). The most frequent CT findings were ground glass opacities (83.3%), subpleural lines (72.2%), traction bronchiectasis (37.5%), and consolidations (31.9%). LUS revealed irregular pleural lines as a common abnormality initially (56.9%), along with subpleural consolidation >2.5 mm ≤10 mm (26.5%) and B-lines (26.5%). A strong correlation was found between LUS score, calculated by artificial intelligence percentage involvement in ground glass opacities described in CT (r = 0.702, *p* < 0.05). LUS score was significantly higher in the group with fibrotic changes compared to the non-fibrotic group with a mean value of 19.4 ± 5.7 to 11 ± 6.6, respectively (*p* < 0.0001). LUS might be considered valuable for examining patients with persistent symptoms after recovering from COVID-19 pneumonia. Abnormalities identified through LUS align with CT scan findings; thus, LUS might potentially reduce the need for frequent chest CT examinations.

## 1. Introduction

During the SARS-CoV-2 pandemic, transthoracic lung ultrasound (LUS) began to be widely used in addition to computed tomography (CT), currently considered the “gold standard” in diagnosing lung lesions in COVID-19 pneumonia [1]. Ultrasonography has primarily been used during the acute phase of the disease within Hospital Emergency Departments and Intensive Care Units, as it allows bedside monitoring without risking transport for patients in critical condition [2].

The radiologic sequelae of COVID-19 pneumonia include ground glass opacities (GGOs), subpleural and interlobular septal thickening, mosaic attenuation of the lung parenchyma, consolidations, reticular opacities, traction bronchiectasis, lung architectural distortion, and honeycombing [3]. The development of pulmonary fibrosis following COVID-19 is a consequence of impaired healing and is unequivocally related to the severe course of the initial phase of the disease [4,5,6,7,8,9,10,11,12,13].

The term “post-COVID-19” is used to describe individuals who, after more than 12 weeks, experience symptoms and complaints resulting from SARS-CoV-2 infection [14]. Special attention should be given to patients whose symptoms and radiological changes do not improve over time and in whom interstitial pulmonary fibrosis develops [15]. According to the British Thoracic Society (BTS) guidelines, it is necessary to perform a physical examination 4 weeks after hospital discharge and a chest X-ray after 12 weeks in patients recovering from COVID-19 pneumonia. If the X-ray shows abnormalities, high-resolution computed tomography (HRCT) is recommended to evaluate interstitial changes in the lungs, or a CT scan with pulmonary artery angiography to rule out pulmonary embolism. If fibrotic changes are observed on the chest CT, referral of the patient to a reference center for interstitial lung disease is necessary [16,17,18,19,20,21,22,23,24,25]. Artificial intelligence (AI), based on deep learning and machine learning algorithms, has been introduced to simplify and expedite the assessment of disease severity on chest CT scans. The result of the analysis is usually presented as the percentage of lung involvement. AI assistance does not replace the radiologist, but rather assists in their work, making it easier to issue final results [26,27,28,29,30].

According to the current literature, the presence of increased B-lines, subpleural consolidations, and a “white lung” image on LUS aligns with areas of ground glass opacities on chest CT [31,32,33,34]. Ultrasound evaluation demonstrated a relatively high level of agreement with CT scans and showed good reproducibility in the acute phase of COVID-19. There is limited literature available on the morphological changes in post-COVID-19 seen in LUS [35,36,37]. Assessing the accuracy of transthoracic lung ultrasound compared to chest CT could provide insights into its utility for disease monitoring.

Thus, the primary objective of this study was to identify and characterize the lung lesions detectable in LUS in post-COVID-19 syndrome. The secondary aim was to describe the association of both CT and LUS and to determine the LUS scoring, which could be used as a screening for persistent lung abnormalities after COVID-19 pneumonia.

## 2. Materials and Methods

This study was approved by the Bioethics Committee at the National Institute of Tuberculosis and Lung Diseases in Warsaw, Poland (approval No. KB-92/2020 dated 10/12/2020), and was conducted in accordance with the Declaration of Helsinki. All qualified patients provided informed consent to participate in this study.

### 2.1. Study Selection

In a single pulmonary department, a prospective, observational study was conducted involving all patients hospitalized from 1 January 2021 to 30 September 2022 who met the following criteria:

(a) Age > 18 years;
(b) Confirmed SARS-CoV-2 infection by antigen testing, reverse transcription polymerase chain reaction (RT-PCR) test of nasopharyngeal swab or positive IgM or IgG SARS-CoV-2 antibodies after the acute phase of the disease in medical history;
(c) Radiologically documented lung lesions consistent with viral pneumonia;
(d) The resolution of the acute phase of the viral pneumonia and persistent clinical symptoms;
(e) The time difference between LUS and chest CT scanning did not exceed 21 days.

Data on the patient’s demographics, comorbidities, smoking history, course of COVID-19 pneumonia, and oxygen saturation were collected from the patient’s medical history. Each patient underwent a chest CT scan and transthoracic ultrasonography as part of their diagnostic assessment of persistent clinical symptoms. Additionally, 35 patients received a second follow-up, while 5 underwent a third examination. In total, 112 chest ultrasound examinations were performed and compared with the corresponding chest CT image.

### 2.2. Lung Ultrasound

Transthoracic lung ultrasound and pleural line measurements were conducted on all patients using an Esaote MyLabSeven Ultrasound System with a linear array transducer (SL1543 3–13 MHz) by a respiratory medicine physician experienced with LUS following current recommendations [38,39,40]. The examination was performed using the recommended image settings for lung ultrasound, and each time, the settings were adjusted individually for each patient to optimize image quality. Artifact-eliminating filters such as noise reduction and harmonic and cross-field imaging were deactivated during the examination. A single focus on the pleural line and image gain was adjusted according to the patient [39,41,42,43,44]. All examinations were recorded on external storage media in both image and video formats.

The examination was performed in the supine and sitting positions depending on the patient’s clinical condition. The anterior and lateral regions of the chest were assessed in the patient’s supine position, while the posterior areas were examined in the sitting position. If, due to severe dyspnea, the patient was unable to lie flat, then the anterior parts of the chest were examined in a semi-sitting position.

**Figure 1:**
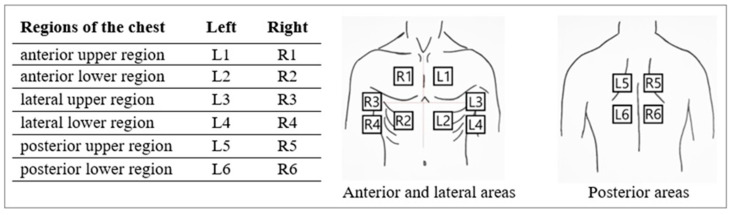
Thoracic regions used for LUS examination protocol. LUS—lung ultrasonography.

According to the examination protocol used, 12 regions of the chest were evaluated in each patient. Each lung was divided into 6 regions to be examined (Figure 1).

In the first examination, the maximum number of lung zones examined was 864-12 areas in 72 patients. In the second follow-up, the maximum number of areas studied was 420-12 areas in 35 patients. 

**Figure 2:**
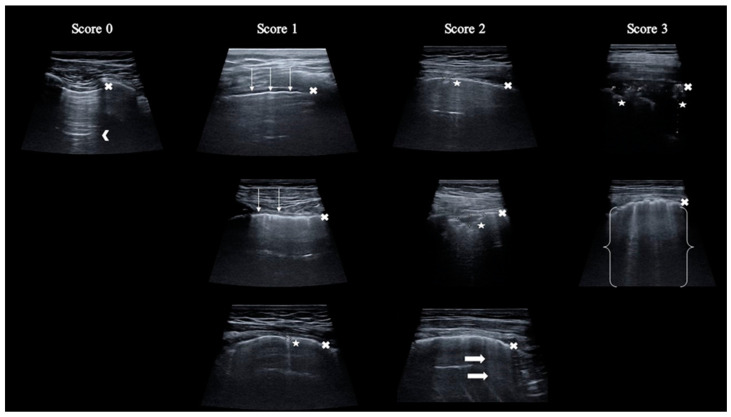
Graphical presentation of LUS scoring scale. Legend: x—pleural line; arrowhead—A-line; thin arrow—irregular pleural line; asterisk—consolidations; thick arrow—B-line; bracket—white lung.

In a specified region, each intercostal space underwent assessment for normal signs and pathological artifacts. Each area was scored, ranging from 0 to 3 points, depending on the pathological lesion described (Table 1 and Figure 2). The total score was calculated by adding up individual scores from each of the 12 regions, with a potential rate of 0 to 36 points. The results of the chest CT scan were blinded to the LUS operator.

### 2.3. Chest CT

Chest CT scans were obtained with a GE-Revolution EVO CT scanner. The imaging protocol was selected individually according to clinical indications. For patients with suspected pulmonary embolism, a CT pulmonary angiogram was performed. The presence of the following abnormalities was analyzed qualitatively by an experienced radiologist in each chest CT scan:

(a) Ground glass opacities;
(b) Mosaic attenuation of the lung parenchyma;
(c) Subpleural lines;
(d) Interlobular septal thickening.

Lung fibrosis was defined based on the following:

(e) Reticulation;
(f) ±Traction bronchiectasis;
(g) ±Honeycombing.

In the case of a contrast-enhanced protocol, the presence of (h) pulmonary embolism was also considered.

Lung and mediastinal reconstructions were evaluated according to a standardized protocol. The CT scans underwent analysis using the Simens Pulmonary Density computer program (AI COVID-19 Plug-in automatic quantification), an artificial intelligence tool that evaluated the extent of involvement of individual lobes and, successively, the entire lungs by areas of ground glass opacities. To characterize overall lung involvement, the program used the Opacity Percentage (the percentage of the predicted volume of abnormal lesions compared to the total lung volume). The calculated results can be used to analyze the disease severity and to monitor the course of lung lesions in COVID-19 pneumonia [45].

### 2.4. Statistical Analysis

Descriptive analyses for quantitative data were evaluated by calculating the mean, median, and standard deviation. For qualitative data, the Chi-square test was used. To evaluate the normality of the data distribution, the Shapiro–Wilk test was performed. Statistical differences in normally distributed data were calculated using Student’s T test, while the non-parametric U-Mann–Whitney test was applied for non-normally distributed data. A Pearson’s linear correlation test was performed between ultrasound scores and CT scan results in Opacity Percentage calculated by AI. The calculated correlation coefficient (r) was assigned as follows: |r| ≤ 0.2—no correlation or very weak correlation; 0.2 < |r| ≤ 0.4—weak correlation; 0.4 < |r| ≤ 0.7—moderate correlation; 0.7 < |r| ≤ 0.9—strong correlation; 0.9 < |r| ≤ 1.0—very strong correlation. Furthermore, two groups of patients were distinguished: one showing signs of fibrosis on chest CT (reticulation, traction bronchiectasis, honeycombing) and another with no indication of fibrosis. A Receiver Operating Characteristic (ROC) curve was plotted to determine LUS’s ability to detect more than 10% of GGOs in chest CT categories (thus indicating the presence or absence of GGOs). An area under the ROC curve (AUC) of lower than 0.6 was considered as showing no discrimination ability, AUC 0.6–0.7 was poor, AUC 0.7–0.8 was moderate, AUC 0.8–0.9 was good, and AUC > 0.9 showed very good/excellent discrimination ability. For all analyses, a *p*-value below 0.05 was considered to be statistically significant.

## 3. Results

### 3.1. Population Characteristics

Seventy-two patients with a history of COVID-19 pneumonia (27 women and 45 men) were included in this study. At the initial patient assessment, oxygen saturation measured by pulse oximeter was a mean of 96 ± 2.34%. Thirteen (18%) patients required oxygen therapy during the first evaluation. Detailed characteristics of the study population are shown in Table 2.

The first evaluation occurred within a mean of 205 days (I e); 35 patients from the initial group were evaluated in the second follow-up, which occurred within a mean of 364 days (II e) after the COVID-19 pneumonia, and the third evaluation occurred within a mean of 495 days (III e) after the onset of COVID-19, which involved five patients from the initial population. 

### 3.2. CT Scan

Ground glass opacities were the most frequently observed radiographic abnormality on CT scans, occurring in 83,3% of examinations during examination I and in 60% during II e. Fibrotic changes such as traction bronchiectasis and honeycombing were present in 30 patients (41.6%) in I e and 11 (31.4%) in II e. In total, the most frequent abnormalities observed in CT were subpleural lines (75%) and GGOs (74.1%). Detailed data of the CT results are shown in Table 3.

The mean extent of lung involvement by ground glass opacities estimated by AI in I e was 16.2 ± 19.3%, whereas it was significantly smaller in II e—4 ± 7.4% (*p* < 0.001). There was no statistical difference between the female and male subgroups. The characteristics of the data are outlined in Table 4.

The extent of GGOs on CT assessed by AI did not correlate with the age of the patients (r = 0.0039, *p* = 0.976; and r = 0.16, *p* = 0.28, respectively), nor did it correlate with smoking history (*p* = 0.93 and *p* = 0.053, respectively) or oxygen therapy use during COVID-19 pneumonia (*p* = 0.49 and *p* = 0.21, respectively). Calculations for III e were not conducted due to the small sample size undergoing this assessment.

### 3.3. Lung Ultrasound

The most frequent lesion detected on lung ultrasound was an irregular pleural line, with a prevalence of 56.9% in I e and 38.3% in II e. Additionally, B-lines were observed in 26.5% (I e) and 14% (II e), while subpleural consolidations > 2.5 mm and ≤10 mm were observed in I e in 26.5% of the cases, with 13.8% in II e. Detailed data on the characteristics of the ultrasound scores and all identified lesions, along with the number of areas involved, are provided in Table 5.

The mean interval between LUS examination and chest CT was 3.2 ± 4.6 days, with a median value of 1 day. The mean score obtained from lung ultrasound in I e was 14 ± 7.4, in II e it was 8.8 ± 5.7, and in III e it was 6.4 ± 4.5. A statistically significantly lower score was evident in II e compared to I e with a *p*-value = 0.000375. The data are shown in Table 6.

### 3.4. LUS-CT Correlation and ROC Curve Model

**Figure 3:**
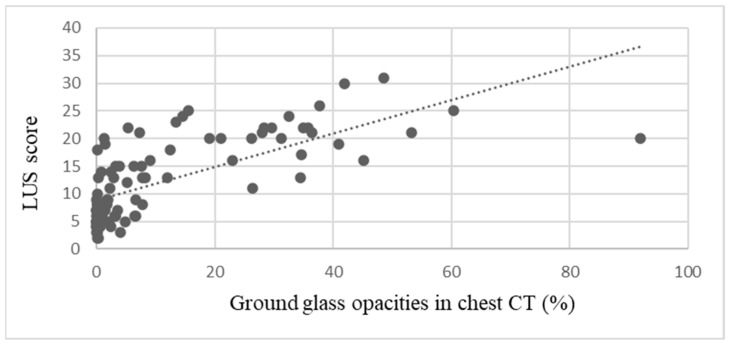
Correlation of LUS score and extent of GGOs in chest CT; r = 0.702; *p* < 0.05.

To establish the correlation between the ultrasound score and the percentage extent of GGOs in the CT scan, as evaluated by AI, Pearson’s linear correlation coefficient was computed. The overall correlation coefficient for all pairs of studies (N = 112) was r = 0.702, *p* < 0.05, indicating a strong positive correlation (Figure 3).

**Figure 4:**
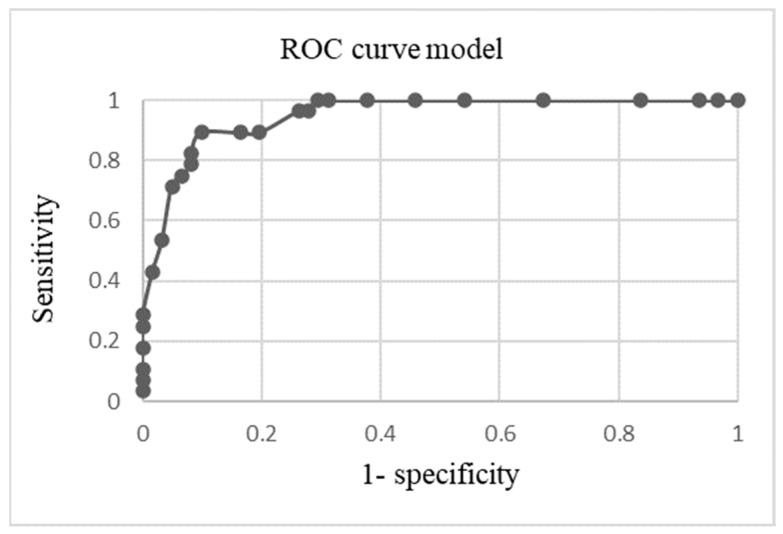
ROC curve model for the utility of LUS score to detect more than 10% of GGOs in chest CT obtained by AI assessment; AUC 0.948 (0.95 Conf. Int.).

An ROC curve was plotted to assess the utility of LUS to detect more than 10% of GGOs in the chest CT. The optimal LUS score for the detection of more than 10% of GGOs based on AI-CT description was 13 points, which combined the highest sensitivity of 0.964 and lowest false-positive rate of 0.262 (specificity 0.738; NPV (negative predictive value) 0.904; PPV (positive predictive value) 0.89). The AUC was calculated as 0.948, indicating excellent discriminatory capability. The above results are shown in Figure 4.

Fibrotic changes were observed in 41.6% of the patients. In both I e and II e, the LUS score was significantly higher in patients with radiological evidence of fibrosis compared to those without (*p* = 0.000002, and *p* = 0.000000, respectively). The mean ultrasound score in I e for the group exhibiting fibrotic features was 19.4 ± 5.7 points, whereas, for those without these features, it was 11 ± 6.6 points, and in II e, these scores were 16 ± 5.3 points and 6 ± 2.7 points, respectively (Table 7). Additionally, all patients with an ultrasound score below nine points showed near complete regression of lung lesions on chest CT.

## 4. Discussion

The COVID-19 pandemic enabled the rapid growth of transthoracic lung ultrasonography worldwide. This low-cost and reproducible test, used alongside CT scanning, was widely employed during the acute phase of the disease.

In studies published to date, the authors have proven that lung ultrasound had high sensitivity (89.5%) and a high negative predictive value (86.6%) for patients suspected of having COVID-19. However, its specificity (70.2%) and positive predictive value (75.5%) may pose challenges, when the incidence of COVID-19 is lower in the future. The presence of similar ultrasound lung lesions in patients with other conditions raises concerns about potential misdiagnosis due to factors such as pulmonary oedema, bacterial pneumonia, viral types of pneumonia such as influenza virus infection, or interstitial pulmonary fibrosis. Given these limitations and considerations about its inability to visualize deeper parts of the lung tissue, it is advisable to interpret lung ultrasound results in relation to additional examinations like clinical evaluation, laboratory tests or a chest CT scan [46,47,48].

This research was a prospective, observational study conducted on a relatively large group of patients. The study included 72 individuals, diagnosed at our department due to persistent clinical symptoms following COVID-19 pneumonia. A total of 112 pairs of chest ultrasound examinations were performed concurrently, with chest CT scans serving as the reference standard. It is noteworthy that all patients had abnormal chest CT scans and a considerable number of patients underwent a second follow-up examination to evaluate the effectiveness of lung ultrasonography in monitoring lung lesions following COVID-19 pneumonia.

To date, there has been no reliable research on transthoracic ultrasonography in patients over 180 days after COVID-19 pneumonia and no published studies detailing the application of transthoracic ultrasonography for monitoring COVID-19 pneumonia lesions alongside chest CT scans. Giovannetti et al. [37] assessed lung ultrasound images using a linear transducer in 38 patients after 3 months following COVID-19, while only visually assessing chest CT images. The study analyzed the correlation between ultrasound and chest CT scans but did not differentiate a group with fibrotic features post-COVID-19 pneumonia. In contrast, Russo et al. [35] performed transthoracic ultrasound and chest computed tomography in 74 patients 6 months after COVID-19 pneumonia using a Convex-type transducer. Meanwhile, Clofent et al. [36] evaluated 352 patients consecutively between the second and fifth month after COVID-19, using ultrasound as a screening test to detect fibrotic-type lesions with a Convex probe.

In our study, the most frequently observed abnormality detected on transthoracic lung ultrasound in both assessments was an irregular pleural line, with occurrences of 56.9% and 38.3%, respectively. Following this, B-lines and subpleural consolidations measuring >2.5 mm and ≤10 mm were reported at rates of 26.5% each during I e, and at lower rates of 14% and 13.8%, respectively, during II e. Giovannetti et al. [37] identified pathologic B-lines in 63.2% of their patients, while Russo et al. [35] visualized an irregular pleural line pattern similarly to our study. They described this artifact in 52.8% of their patients, along with subpleural consolidations < 1 cm seen in 43% of individuals. In Clofent et al.’s study [36], the most commonly evaluated lesion was the B-line (53%).

In the present study, the mean percentage of ground glass opacities in chest CT scans in I e averaged 16.2% ± 19.3%, while in II e this was significantly lower at 4% ± 7.4% (*p* < 0.001). Similar results were observed in the analysis of LUS score results, suggesting the resolution of some lung lesions over the follow-up period on LUS. However, complete resolution was not achieved in either chest computed tomography evaluations or transthoracic ultrasound examinations as no patient had a normal image or scored zero on the ultrasound score.

The prevalence of the radiological findings in the chest CT scans was similar to that reported by Russo et al. [35], with ground glass opacities being the most frequently described abnormality (in 83.3% of cases in I e and 60% of cases in II e), followed by subpleural lines (72.2% and 82.8%, respectively). Architectural distortion and fibrotic lesions such as traction bronchiectasis and honeycombing were identified in 41.6% of patients during I e and 31.4% during II e.

The results of our research indirectly indicate that lung abnormalities detected through ultrasound and chest CT scan continue to persist long after the acute phase of COVID-19 pneumonia, regardless of its severity. Watanabe et al.’s [49] meta-analysis of 3134 cases showed that a significant number of patients (32.6%) exhibited persistent chest CT changes one year post-COVID-19 pneumonia. The most prevalent changes included ground glass opacities and fibrotic changes (21.2% and 20.6% of patients, respectively).

One of the secondary objectives of this research was to evaluate the relationship between abnormalities observed in LUS and the percentage of GGOs identified on chest CT scans by AI. The Pearson linear correlation coefficient calculated for all 112 pairs of ultrasound and chest CT scans revealed a strong positive correlation.

Additionally, the percentages of GGOs in CT were lower in consecutive examinations, and a statistically significantly lower LUS score was evident in II e compared to I e, which indirectly suggests that improvements in CT lesions could align with decreased scores obtained from follow-up ultrasound examinations. Furthermore, in cases where the ultrasound score is below nine points, the chest CT scan indicated a significant regression of COVID-19 lesions, leaving small discrete areas of ground glass opacities and post-inflammatory lesions, which indicate that those patients may not require further CT scans. On the contrary, an ultrasound score exceeding 13 in a new patient might facilitate the early detection of pulmonary abnormalities, allowing for timely intervention. LUS might be used for regular monitoring of lung abnormalities in post-COVID-19 patients, potentially reducing the need for repeated CT scans and associated radiation exposure. However, these conclusions warrant more extensive investigations involving a larger patient cohort. Currently, it remains uncertain if solely using lung ultrasound is sufficient for monitoring such lesion dynamics.

In order to determine if lung ultrasound could be used to screen fibrotic changes in post-COVID-19 patients, we compared the LUS scores in two groups of patients: with and without fibrotic changes in CT. Some publications discuss the use of transthoracic lung ultrasonography to assess interstitial lung fibrosis among patients with systemic connective tissue diseases (CTDs) presenting with lung involvement [50,51,52,53]. The diagnostic gold standard is HRCT, but it exposes individuals (often young) to ionizing radiation. Lung ultrasound assesses the presence of B-lines observed during an examination and compares them with the Warrick scale, used to evaluate the severity of lung lesions on a CT scan. In a meta-analysis by Song et al. [54], the sensitivity of LUS ranged between 73.5% and 100%, while the specificity ranged from 56% to 100%. The overall sensitivity across all studies analyzed was found to be 91.5%, with a specificity of 81.3%. However, these findings were limited to systemic scleroderma cases where the Warrick scale had been validated. The study concluded that lung ultrasonography holds high diagnostic accuracy and correlates well with CT scan images depicting identified lesions. The number of B-lines in LUS compared with the Warrick scale in CT shows a significant correlation. Similar conclusions were obtained from studies in patients with idiopathic pulmonary fibrosis [55,56,57].

In our research, the LUS score showed a statistically significant increase in patients with fibrotic features on chest CT in both I e and II e. Similar results were presented by Clofent et al. [36]. In their study, Giovannetti et al. [37] estimated the sensitivity of ultrasound for detecting interstitial abnormalities (including GGOs) in the lungs based on the ROC curve at 0.81 with a false-positive rate of 0.25. It should be noted that the authors used a different scoring scale and the patients were evaluated 3 months after having COVID-19, which may have affected the difference in results.

Despite limited scientific reports on the morphological changes observed in lung ultrasound examinations over longitudinal follow-up, the findings of this study show similarity to those presented in the previously referenced works. It appears that lung ultrasound examinations may serve as a novel tool for detecting and monitoring the dynamics of radiological changes associated with COVID-19. LUS is convenient, cost-effective, widely accessible, repeatable, and, importantly, does not subject patients to ionizing radiation. It can be easily conducted in an outpatient setting and functions as a screening test for assessing post-COVID-19 pneumonia lung abnormalities. Additionally, these results yield supplementary insights into potential applications in other respiratory conditions. If there exists a correlation between reduced ultrasound scores and the regression of ground glass opacities observed in computed tomography, then this method might aid in monitoring various interstitial diseases manifesting with ground glass opacities or their responsiveness to treatment. Furthermore, it might offer early assessment capabilities and screening for fibrosis; however, further research on appropriately selected patient groups would be required to explore this aspect thoroughly.

## 5. Conclusions

Based on the study findings, lung ultrasound should be considered as a diagnostic method for post-COVID-19 patients experiencing persistent clinical symptoms. The results of transthoracic lung ultrasonography may indicate the presence of radiological signs of lung fibrosis, but further research is required.

## 6. Strengths and Limitations

This study presents several noteworthy strengths. This research is the longest observation of lung abnormalities described in LUS published for post-COVID-19 follow-up and was conducted prospectively and involved a comparison with the widely accepted chest CT scan. A specific scoring system was utilized, and a sizable number of patients were enrolled in the study. An automated computer program assessed the CT scan, thereby removing any subjective variances in radiological visual interpretation. Additionally, both the operator conducting the ultrasound examination and the radiologist evaluating the CT results were blinded to the results of the corresponding method. 

Despite utilizing the same examination schedule for all patients, only a subset of them underwent an LUS on the same day as their chest CT. In other cases, this interval was longer but did not exceed 21 days, potentially impacting the results. It is important to highlight that the median interval between these two examinations was just one day. This study was conducted at a single center and all LUS assessments were carried out by one operator, precluding the assessment of interobserver agreement. The discrepancy in the number of patients subjected to the second and third analysis precluded more accurate analyses. Furthermore, it is crucial to note that neither the author’s transthoracic lung ultrasound scoring scale nor the computer program used for objectively determining GGOs in chest CT had been validated in a large prospective clinical trial prior to this study. However, none of these limitations appear to affect the main objective.

## 7. Future Aspects

Despite the promising findings regarding the potential of LUS in post-COVID-19 care, its clinical utility and optimal integration into routine practice remain uncertain. Large-scale, multicenter studies with longer follow-up periods are needed to establish evidence-based guidelines and protocols for the use of LUS in post-COVID-19 follow-up. Including patients from multiple centers across different geographical regions and healthcare systems could help mitigate the selection bias. Additionally, incorporating advanced imaging techniques, such as elastography or contrast-enhanced ultrasound, may provide complementary information and improve the diagnostic accuracy of LUS. To validate the findings of LUS studies, future research should consider comparing LUS with more sensitive imaging modalities, such as magnetic resonance imaging (MRI). Furthermore, integrating Weighted Gene Co-Expression Network Analysis (WGCNA) with machine learning validation and explainable artificial intelligence techniques could provide deeper insights into the molecular mechanisms underlying post-COVID-19 lung abnormalities. Transcriptional profiling from bronchial epithelium cells, machine learning validation, and explainable AI methods could elucidate the complex relationships between molecular signatures and post-COVID-19 lung disease manifestations, offering potential targets for personalized treatment strategies [58].

## Figures and Tables

**Table 1:** The scoring scale used in transthoracic lung ultrasound.

Score	Description
**0**	Regular and continuous pleural line, presence of A-lines
**1**	Irregular or broken pleural line, or consolidation ≤ 2.5 mm, or ≤3 B-lines
**2**	Consolidation > 2.5 mm ≤10 mm, or >3 B-lines
**3**	Consolidation > 10 mm, or pleural effusion, or coalescence B-lines, or “white lung” image

**Table 2:** Characteristics of the study population.

Factor	
**Sex, N (%)**	
Female	27 (37.5)
Male	45 (62.5)
**Age (years)**	
Mean ± SD	58 ± 12.9
Median	58 (32–82)
**Comorbidities, N (%)**	
Hypertension	38 (53)
Diabetes mellitus	14 (19)
Asthma	8 (11)
Benign prostatic hyperplasia	7 (9.7)
Atrial fibrillation	7 (9.7)
Heart insufficiency	5 (6.9)
Dyslipidaemia	5 (6.9)
Thyroid disease	5 (6.9)
Coronary artery disease	4 (5.5)
Hiperurycemia	4 (5.5)
**Smoking history, N (%)**	
Smoker	32 (44.4)
Non-smoker	40 (55.6)
**Oxygen therapy during COVID-19, N (%)**	
Passive oxygen therapy	20 (27.6)
High-flow nasal oxygen therapy	10 (13.8)
Non-invasive ventilation	1 (1.4)
Mechanical ventilation	2 (2.8)
Extracorporeal membrane oxygenation	1 (1.4)
**Testing for COVID-19, N (%)**	
RT-PCR test	51 (70.8)
Antigen testing	13 (18.1)
Increase in anti-SARS-CoV-2 antibody titers	8 (11.1)
**Clinical symptoms at admission, N (%)**	
Dyspnoea	51 (70.8)
Cough	30 (41.6)
Chest pain	14 (19.4)

N, number; SD, standard deviation; COVID-19, coronavirus disease 2019; SARS-CoV-2, severe acute respiratory syndrome coronavirus 2; RT-PCR, reverse transcription polymerase chain reaction.

**Table 3:** Chest CT imaging results.

Abnormality	Examination I	Examination II	Examination III
	N, (%)	N, (%)	N, (%)
Pulmonary embolism	14 (19.4)	0 (0)	0 (0)
Ground glass opacities	60 (83.3)	21 (60)	2 (40)
Consolidations	23 (31.9)	4 (11.4)	1 (20)
Subpleural lines	52 (72.2)	29 (82.8)	3 (60)
Septal thickening	8 (11.1)	1 (2.8)	0 (0)
Mosaic attenuation	19 (26.3)	9 (25.7)	0 (0)
Traction bronchiectasis	27 (37.5)	11 (31.4)	1 (20)
Honeycombing	3 (4.1)	0 (0)	0 (0)

**Table 4:** Characteristics of AI analysis of chest CT scans.

Percentage of GGOs	Examination I	Examination II
**Mean ± SD**	16.2 ± 19.3%	4 ± 7.4%
**Median (range)**	7.2 (0–91.9)%	0.8 (0–36.5)%

SD, standard deviation; GGOs, ground glass opacities.

**Table 5:** Characteristics of LUS.

Lesions in LUS	Examination I	Examination II
	N of Lung Zones, (%)	N of Lung Zones, (%)
Consolidation ≤ 2.5 mm	41 (4.7)	35 (8.3)
Consolidation > 2.5 mm ≤10 mm	229 (26.5)	58 (13.8)
Consolidation > 10 mm	70 (8.1)	7 (1.6)
Irregular pleural line	492 (56.9)	161 (38.3)
Broken pleural line	26 (3)	10 (2.3)
B-line	229 (26.5)	59 (14)
“White lung”	15 (1.7)	0 (0)
Pleural effusion	9 (1)	5 (1.1)

N, number; LUS, lung ultrasound.

**Table 6:** Characteristics of LUS scores.

LUS Score	Examination I	Examination II	Examination III
**Mean ± SD**	14 ± 7.4	8.8 ± 5.7	6.4 ± 4.5
**Median (range)**	13.5 (2–31)	7 (2–23)	6 (0–12)

SD, standard deviation; LUS, lung ultrasound.

**Table 7:** Characteristics of LUS scores in two subgroups of patients.

Mean LUS Score	Examination I	Examination II	
**Fibrotic changes present**	19.4 ± 5.7	16 ± 5.3	*p* = 0.000002
**Without fibrotic changes**	11 ± 6.6	6 ± 2.7	*p* = 0.000000

LUS, lung ultrasound.

## Data Availability

Requests to access the datasets should be directed to the corresponding author.
